# Supplementary material for: Reliability and reproducibility of an Italian questionnaire on “Knowledge of high social impact Eye Diseases” (KED-IT)
Source: PeerJ. 2024 Oct 3;12:e17906. doi: 10.7717/peerj.17906 (PMC11456289; doi:10.7717/peerj.17906)
Supplement: Supplemental Information 2 [file peerj-12-17906-s002.docx]

**DATASET - English-language codebook**

**SOGGETTO**: subject identifier number

**GENERE**: Gender at birth: 0 = male 1 = female Età: age years

**T. STUDIO**: Education:0 = elementary 1 = middle school 2 = high school 3 = bachelor or upper

**@1a:** In the past year, have you received information about eye diseases? 0 = No/ I don't remember 1 =Yes

**@2a:** If so, from what source of information? (indicate the main one): 0 = Magazines/newspapers ­ 1 = Educational pamphlets ­ 2 = Television ­ 3 = Radio ­ 4 = Internet/social media ­ 5= Social or religious organizations ­ 6 = At a doctor’s office, clinic, or community health screening ­ 7 = At your workplace 8 = From relatives or friends

**newa3:** 1st item KEDIT (see table1 in the manuscript) at T0 (1st administration): 0 = wrong answer/I don't know; 1 = correct answer.

**newa4:** 2nd item KEDIT (see table1 in the manuscript) at T0 (1st administration): 0 = wrong answer/I don't know; 1 = correct answer.

**newa5:** 3rd item KEDIT (see table1 in the manuscript) at T0 (1st administration): 0 = wrong answer/I don't know; 1 = correct answer.

**newa6:** 4th item KEDIT (see table1 in the manuscript) at T0 (1st administration): 0 = wrong answer/I don't know; 1 = correct answer.

**newa7:** 5th item KEDIT (see table1 in the manuscript) at T0 (1st administration): 0 = wrong answer/I don't know; 1 = correct answer.

**newa8**: 6th item KEDIT (see table1 in the manuscript) at T0 (1st administration): 0 = wrong answer/I don't know; 1 = correct answer.

**newa9:** 7th item KEDIT (see table1 in the manuscript) at T0 (1st administration): 0 = wrong answer/I don't know; 1 = correct answer.

**newa10:** 8th item KEDIT (see table1 in the manuscript) at T0 (1st administration): 0 = wrong answer/I don't know; 1 = correct answer.

**newa11:** 9th item KEDIT (see table1 in the manuscript) at T0 (1st administration): 0 = wrong answer/I don't know; 1 = correct answer.

**newa12:** 10th item KEDIT (see table1 in the manuscript) at T0 (1st administration): 0 = wrong answer/I don't know; 1 = correct answer.

**newa13:** 11 th item KEDIT (see table1 in the manuscript) at T0 (1st administration): 0 = wrong answer/I don't know; 1 = correct answer.

**newa14:** 12 t h item KEDIT (see table1 in the manuscript) at T0 (1st administration): 0 = wrong answer/I don't know; 1 = correct answer.

**newa15:** 13th item KEDIT (see table1 in the manuscript) at T0 (1st administration): 0 = wrong answer/I don't know; 1 = correct answer.

**Newa16:** 14th item KEDIT (see table1 in the manuscript) at T0 (1st administration): 0 = wrong answer/I don't know; 1 = correct answer.

**@1b:** T1 (2nd administration): In the past year, have you received information about eye diseases? 0 = No/ I don't remember 1 =Yes

**@2b:** T1 (2nd administration): If so, from what source of information? (indicate the main one): 0 = Magazines/newspapers ­ 1 = Educational pamphlets ­ 2 = Television ­ 3 = Radio ­ 4 = Internet/social media ­ 5= Social or religious organizations ­ 6 = At a doctor’s office, clinic, or community health screening ­ 7 = At your workplace 8 = From relatives or friends

**newb3:** T1 (2nd administration): 1st item KEDIT (see the table1 in the manuscript): 0 = wrong answer/I don't know; 1 = correct answer.

**newb4:** T1 (2nd administration): 2nd item KEDIT (see the table1 in the manuscript): 0 = wrong answer/I don't know; 1 = correct answer.

**newb5:** …

**newb6**: …

**newb7:** …

**newb8:** …

**Newb9**: …

**newb10**: …

**newb11**: …

**newb12**: …

**newb13: …**

**newb14: …**

**newb15: …**

**newb16: …**

**NOTE**: comments of the subjects (text)

**scoretot**: score of knowledge KEDIT at T0 range from 0 to 14 Gruppo UL: Group high knowledge Low=0; upper =1
